# Environmental exposure to glyphosate does not inhibit human acetylcholinesterase and butyrylcholinesterase

**DOI:** 10.2478/aiht-2024-75-3822

**Published:** 2024-03-29

**Authors:** Dora Kolić, Vesna Pehar, Zrinka Kovarik

**Affiliations:** Institute for Medical Research and Occupational Health, Division of Toxicology, Zagreb, Croatia; Dr Franjo Tuđman Croatian Defence Academy, Zagreb, Croatia; University of Zagreb Faculty of Science, Zagreb, Croatia

**Keywords:** AChE, BChE, cholinesterase, inhibition, herbicide, neurotoxicity, organophosphorus compounds, pesticide, AChE, BChE, herbicid, inhibicija, kolinesteraza, konstanta disocijacije, neurotoksičnost, organofosforni spojevi

## Abstract

Glyphosate has remained the leading herbicide on the global market to date, despite the continuous debate between consumers, scientific community, and regulatory agencies over its carcinogenicity, genotoxicity, environmental persistence, and the role in the development of neurodegenerative disorders. Chemically, glyphosate belongs to a large family of organophosphorus pesticides, which exert a neurotoxic effect by inhibiting acetylcholinesterase (AChE) and butyrylcholinesterase (BChE), enzymes of the cholinergic system essential for maintaining neurotransmission. Although research shows that glyphosate is a weak cholinesterase inhibitor in fish and mammals compared to other OP compounds, no conclusive data exist concerning the inhibition of human AChE and BChE. In our study we analysed its inhibitory potency on human AChE and BChE, by establishing its IC_50_ and reversible inhibition in terms of dissociation inhibition constants. Glyphosate concentration of 40 mmol/L caused near total inhibition of enzyme activity (approx. 10 % activity remaining). Inhibition dissociation constants (*K*_i_) of glyphosate-AChE and -BChE complexes were 28.4±2.7 mmol/L and 19.3±1.8 mmol/L, respectively. In conclusion, glyphosate shows a slight binding preference for BChE but exhibits inhibition only in a high concentration range. Our results are in line with studies reporting that its neurotoxic effect is not primarily linked to the cholinergic system.

## Abstract (hr)

Glifosat je vodeći herbicid na današnjem svjetskom tržištu, unatoč neprestanim raspravama između potrošača, znanstvene zajednice i regulatornih agencija o njegovoj kancerogenosti, genotoksičnosti, postojanosti u okolišu i utjecaju na razvoj neurodegenerativnih bolesti. Kemijski gledano, glifosat pripada velikoj obitelji pesticida, organofosfornim spojevima (OP) koji imaju neurotoksični učinak inhibirajući acetilkolinesterazu (AChE) i butirilkolinesterazu (BChE), esencijalne enzime kolinergičnoga sustava koji održava proces prijenosa živčanih impulsa. Iako su različita istraživanja pokazala da je glifosat slab inhibitor kolinesteraza u riba, sisavaca i ljudi u odnosu na druge organofosforne spojeve, još uvijek ne postoje konačni podatci o njegovoj inhibiciji ljudske AChE i BChE. U ovoj smo studiji analizirali inhibitorni potencijal za ljudsku AChE i BChE, procijenili IC_50_ vrijednosti i utvrdili reverzibilnu inhibiciju pomoću vrijednosti konstanti disocijacije inhibitora. Glifosat je gotovo u cijelosti inhibirao aktivnost enzima pri 40 mmol/L koncentraciji (preostalo je otprilike 10 % enzimske aktivnosti). Konstante disocijacije (*K*_i_) kompleksa glifosat-AChE i -BChE iznose 28.4±2.7 mmol/L, odnosno 19.3±1.8 mmol/L. Zaključno, glifosat iskazuje malu sklonost za vezanje BChE, no pokazuje inhibiciju u rasponu visokih koncentracija. Rezultati ovoga istraživanja podupiru rezultate postojećih studija prema kojima neurotoksični učinak glifosata nije primarno vezan za kolinergični sustav.

Since its commercialisation in 1974 by Monsanto and the introduction of genetically modified glyphosate-resistant crops in 1996 (1), the extensive use of glyphosate has culminated as it took the leading on the global herbicide market. Glyphosate, *N*-(phosphonomethyl)glycine (Figure 1), is the active ingredient in glyphosate-based herbicides (GBHs) which inhibits 5-enolpyruvylshikimate-3-phosphate synthase (EPSPS), an enzyme unique to plants and microorganisms and crucial for the synthesis of aromatic amino acids tryptophan, tyrosine, and phenylalanine (2). GBHs most often contain 40–60 % of glyphosate in isopropyl ammonium salt form, and the rest of the mixture is composed of water, heavy metals (i.e., arsenic and cobalt), surfactants, usually from the polyoxyethylenamine (POEA) family, and other adjuvants (3). With widespread and unchecked application of GBHs in non-crop situations such as on roadsides, around railway tracks, in pre- and post-cropping of fields, as well as for control of vegetation under the canopy of trees and orchard crops, glyphosate is predictably found in off-target locations and diverse ecosystems (1). In the European Union (EU) 21 % of tested soil contains glyphosate and 42 % its metabolite aminomethylphosphonic acid (AMPA), reaching maximum concentrations of 2 mg/kg (4).

**Figure 1:**
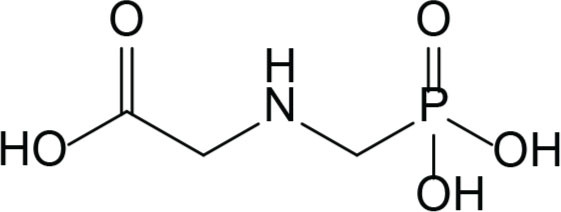
Chemical structure of glyphosate

Although glyphosate has been approved for use by the United States Environmental Protection Agency (US EPA) and the European Food Safety Authority (EFSA), it came under increased scrutiny in 2015, when the International Agency for Research on Cancer (IARC) categorised glyphosate as a group 2A carcinogen (5). This debate is still in progress, as data both support and deny glyphosate carcinogenicity, mostly depending on whether the studies employed the glyphosate salt or a full GBH formulation. A large body of *in vitro*, *in vivo*, and epidemiological evidence, summarised in a paper by Lacroix and Kurrasch (6), shows various toxicities of glyphosate and GBHs across animal species. However, many of the reviewed studies used high glyphosate concentrations which are not detected in the environment or human body, where they range from 10 ng/L to 10 mg/L (approximately 0.591 nmol/L to 59.15 µmol/L) in water ecosystems (7) and 0.16–7.6 µg/L in the urine of the general human population (8). Also, it is difficult to clearly distinguish glyphosate from GBH effects, as GBH components are not publicly disclosed. A comparison of various glyphosate formulations showed difference in cytotoxicity on a human liver cell line that varied as much as 200-fold (3).

Considering its chemical structure, herbicide glyphosate belongs to a large family of pesticides which are organophosphate (OP) compounds. OPs inhibit the activity of the enzyme acetylcholinesterase (AChE) and its related enzyme butyrylcholinesterase (BChE) by phosphorylating the serine of the catalytic triad and forming a stable OP-enzyme conjugate. Inhibition prevents the hydrolysis of acetylcholine (ACh), an important excitatory neurotransmitter, and leads to ACh accumulation in both the peripheral and central nervous system (9). The resulting neurotoxic effects are caused by uncontrolled nerve impulse transmission, which in severe cases of acute poisoning can result in seizures, respiratory failure, and death (9, 10). Furthermore, chronic exposure to OPs below the threshold for acute cholinergic toxicity, often observed in agricultural workers and pesticide sprayers, causes various neurological and cognitive abnormalities known as chronic OP-induced neuropsychiatric disorders (10).

Neurotoxicity effects have been reported in animal models, including a drop in AChE activity in blood and various tissues (11,12,13). However, since pesticides can affect gene expression, reduced AChE activity in homogenates may not only be related to enzyme inhibition but may also result from lower enzyme levels. Thus, the main aim of this research was to evaluate the direct *in vitro* inhibitory effect of glyphosate on human AChE and BChE in terms of dissociation constants and binding selectivity and to confirm our recent results and an estimation that glyphosate is a weak inhibitor of AChE compared to other OP pesticides (14, 15). In this study we evaluated the inhibitory potency of glyphosate concentrations ranging between 1 and 40 mmol/L on human recombinant AChE and human BChE isolated from plasma.

## MATERIALS AND METHODS

### Chemicals and enzymes

Analytical grade glyphosate (Sigma-Aldrich, St. Louis, MO, USA) with a declaration of purity of 99.7 % was a generous gift from Dr Davor Želježić and Dr Vilena Kašuba (Institute for Medical Research and Occupational Health, Zagreb, Croatia). Stock solution (100 mmol/L) was prepared in sodium phosphate buffer solution (0.1 mol/L, pH 7.4) and further dilutions were made in buffer just before use.

Acetylthiocholine iodide (ATCh), thiol reagent 5,5′-dithiobis(2-nitrobenzoic acid) (DTNB), and bovine serum albumin (BSA) were purchased from Sigma-Aldrich. Stock solution of ATCh was prepared in water, while BSA and DTNB were prepared in sodium phosphate buffer (0.1 mol/L, pH 7.4).

Recombinant human AChE and purified human plasma BChE were a generous gift from Dr Florian Nachon (Armed Forces Biomedical Research Institute, Department of Toxicology and Chemical Risk, Bretigny-sur-Orge, France) and were stored at 4 °C before use.

### Reversible inhibition with glyphosate

Reversible AChE and BChE inhibition was measured by determining the decrease in enzyme activity towards substrate ATCh in the presence of a wide range of glyphosate concentrations ensuring 10–90 % inhibition. Enzyme activity was measured following a previously described procedure (16) and assayed with Ellman’s assay (17), where the inhibition mixture contained sodium phosphate buffer, enzyme (AChE or BChE), glyphosate (1–40 mmol/L), DTNB (0.3 mmol/L), and ATCh (0.1–0.7 mmol/L) to start the reaction. 0.01 % BSA was added to the buffer for all measurements containing AChE. Measured activity in the presence of glyphosate was corrected for spontaneous non-enzymatic hydrolysis of ATCh. The assay was performed at 25 °C in 96-well plates on the Infinite M200PRO plate reader (Tecan Austria GmbH, Salzburg, Austria). The dissociation constants of inhibition, *K*_i_, were determined from at least three experiments as described previously (16) using the Prism9 software (GraphPad, San Diego, CA, USA).

The same data and the same software were used to approximate IC_50_ values from a nonlinear fit of the glyphosate concentration logarithm values vs the percentage of enzyme activity.

## RESULTS AND DISCUSSION

**Figure 2:**
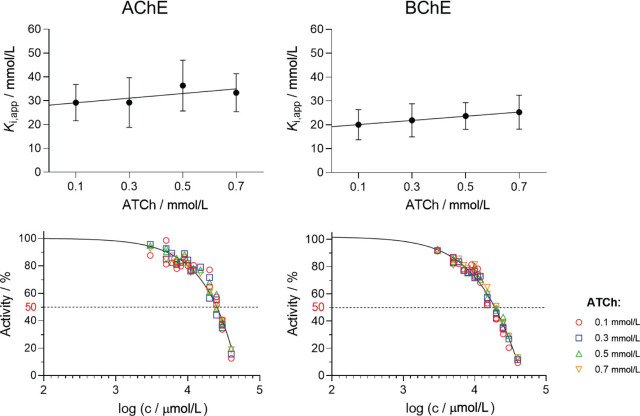
Glyphosate inhibition profiles for human AChE and BChE shown as the dissociation constant of the enzyme-inhibitor complex (*K*_i_, upper panels) and IC_50_ values (lower panels) determined in the presence of substrate acetylthiocholine (ATCh) over a range of glyphosate concentrations (*c*). The same dataset was used to determine both kinetic profiles. Inhibition was evaluated from four experiments measured at 25 °C

Exposure to environmentally relevant glyphosate levels, presumably not harmful to humans, seems to have different effects from exposure to much higher glyphosate doses. Milić et al. (12) reported that low doses produced significant primary DNA damage and inhibited AChE but not BChE in glyphosate-exposed rats, even without increased markers of oxidative stress. On the other hand, Larsen et al. (18) reported that glyphosate was a weak inhibitor of AChE in rats. Glyphosate showed a low potency to inhibit AChE in electric eel (*Electrophorus electricus*), as the inhibition was determined only at higher concentrations, namely 4.5 % and 11 % at 0.75 mmol/L and 1 mmol/L, respectively (13). In equine serum, glyphosate was not able to inhibit BChE at concentrations up to 1 mmol/L (13). While some research suggests that glyphosate inhibits about 20 % of erythrocyte AChE at 5 mmol/L after 4 h of incubation (11), the IC_50_ in human serum is estimated to be 714 mmol/L (19), which is much higher than blood concentrations associated with indirect exposure (<0.05 mmol/L) or acute poisoning (0.05–5.0 mmol/L) (11). Here we determined that human AChE and BChE have a similar binding affinity (1/*K*_i_) for glyphosate and that glyphosate reversibly inhibits both cholinesterases in the lower millimolar range (Figure 2, Table 1) with a slight binding preference for BChE. The inhibition profiles of the upper panels in Figure 2 show similar *K*_app_ for all tested substrate concentrations, indicating a non-competitive mode of inhibition. We used the same experimental data to approximate the IC_50_ values of glyphosate for both cholinesterases (Figure 2, lower panels). As ATCh concentration did not significantly influence the degree of inhibition, we determined a joint IC_50_ value for each enzyme by roughly projecting the point of inflection of the curve with the line representing 50 % of enzyme activity inhibition on the x-axis. The approximated IC_50_ values (Table 1) of 25.1 mmol/L for human AChE and 20.0 mmol/L for human BChE do not significantly differ from the dissociation constants of inhibition. It is worth highlighting that we checked glyphosate ability to bind both enzymes covalently, and we can confirm that glyphosate is not a progressive inhibitor of cholinesterase, even though it is an organophosphate. Namely, only organophosphates with fully substituted phosphorus atom have a potential for the nucleophilic attack on the catalytic serine that results with conjugation and consequently with progressive inhibition of cholinesterases.

**Table 1:** Dissociation constants (*K*_i_) of glyphosate for human AChE and BChE and approximated IC_50_ values

**Enzyme**	***K*_i_ (mmol/L)**	**Approx. IC_50_ (mmol/L)**
AChE	28.4±2.7	25.1
BChE	19.3±1.8	20.0

Concerning acute poisonings, a case study of a patient who ingested a glyphosate-based herbicide and developed an intermediate-like neurotoxicity syndrome revealed a decrease in serum levels of BChE (20). However, according to our results and other studies, cholinesterase inhibition seems unlikely to be a mechanism of neurotoxicity (10, 21, 22). In other words, the capacity of glyphosate to induce oxidative stress, neuroinflammation, and mitochondrial dysfunction, processes that lead to neuronal death by autophagy, necrosis, or apoptosis (23), or to induce behavioural and motor disorders is not likely to be a consequence of inhibited AChE activity. One study on human neuroblastoma SH-SY5Y cells (24) reports that glyphosate and its main metabolite, aminomethylphosphonic acid (AMPA), are cytotoxic and neurotoxic for neuronal development via oxidative stress and induce neurite outgrowth, apoptosis, autophagy, and necrotic signalling pathways.

While *in silico* analyses imply poor glyphosate ability to pass the blood-brain barrier (BBB) (15), a systematic review by Costas-Ferreira et al. (23) clearly documents the neurotoxicity and mechanisms of action of glyphosate in the nervous system of various animal species and humans. According to Martinez and Al-Ahmad (25), it seems that both glyphosate and AMPA can increase BBB permeability, possibly by interfering with the proteins that mediate hermetic junctions between BBB endothelial cells. This study also showed that glucose uptake by brain endothelial cells increased after exposure to high doses of glyphosate.

Glyphosate also seems to exert a significant toxic effect on neurotransmission, with the glutamatergic system being one of the most affected systems (24,25,26,27,28). Intranasal administration of glyphosate has been reported to reduce the number of cholinergic neurons, which was evidenced by lower expression of choline acetyltransferase (ChAT), the enzyme responsible for the synthesis of neurotransmitter ACh, as well as of the alpha-7 nicotinic ACh receptor (α7-nAChR) in the hippocampus (29). These effects could be responsible, at least in part, for anxiety, memory deficit and locomotor disturbances (30), as well as for lower body weight gain and depression-like behaviour, which implies the dopaminergic and serotoninergic system impairment (31). In addition, one study (32) has showed that glyphosate can infiltrate the brain, elevate the expression of tumour necrosis factor alpha (TNFα) and soluble amyloid beta (Aβ), and disrupt the transcriptome in a dose-dependent manner.

## CONCLUSION

Glyphosate is a weak inhibitor of both human AChE and BChE. In other words, environmental exposure to glyphosate, which is in the micromolar range, does not inhibit acetylcholinesterase. Inhibition occurs only at very high, 1000-fold doses.
